# PRIMARY CARE PROVIDER PERSPECTIVES ON LANGUAGE AND COMMUNICATION BARRIERS IN DEMENTIA DIAGNOSIS AND CARE

**DOI:** 10.1093/geroni/igac059.2794

**Published:** 2022-12-20

**Authors:** Alma Hernandez de Jesus, Melissa Ma, Daniel Dohan, Cecilia Alagappan, Katherine Rankin, Alissa Sideman

**Affiliations:** UCSF, San Francisco, California, United States; University of California, San Francisco, San Francisco, California, United States; UC San Francisco, San Francisco, California, United States; University of California San Francisco, Memory and Aging Center, San Francisco, California, United States; University of California San Francisco, San Francisco, California, United States; University of California, San Francisco, San Francisco, California, United States

## Abstract

Dementia diagnosis and care relies on extensive communication between a doctor, patient, and oftentimes a family caregiver. Communication is important for recognizing when there is a cognitive concern, gaining an understanding of the patient’s history of cognitive decline, engaging in cognitive testing, and providing quality care post-diagnosis. We conducted a qualitative study of 35 primary care clinicians and primary care nurse practitioners working in safety net settings in California to understand facilitators and barriers to dementia diagnosis and care. Using thematic analysis, we identified similar themes to those that have been explored extensively related to language and communication in doctor-patient relationship. Topics ranging from challenges that emerge when there is language discordance, difficulties finding and using interpreters, and challenges related to communicating medical topics in a patient’s native language. Specifically related to dementia, we found that language challenges emerge due to inadequate translation or availability of cognitive testing and post-diagnostic resources in multiple languages. However, we also identified unique challenges related to communication, including hearing loss, communicating about trauma during the diagnostic history interview, navigating the logistics of care, including communicating with specialists, building trust and rapport when there is language discordance, and cultural miscommunication even when the spoken language is the same. These issues affect historically marginalized individuals and communities, especially when dementia and cognitive impairment are present. We suggest new approaches and policies to enhance communication and better ways of working with patients who are hard of hearing.

